# Infant feeding experiences among Indigenous communities in Canada, the United States, Australia, and Aotearoa: a scoping review of the qualitative literature

**DOI:** 10.1186/s12889-024-19060-1

**Published:** 2024-06-13

**Authors:** Hiliary Monteith, Carly Checholik, Tracey Galloway, Hosna Sahak, Amy Shawanda, Christina Liu, Anthony J. G. Hanley

**Affiliations:** 1 Department of Nutritional Sciences, Temerty Faculty of Medicine, University of Toronto, University of Toronto Medical, King’s College Circle, Sciences Building, 5th Floor, Room 5253A, Toronto, ON M5S 1A8 Canada; 2 Department of Anthropology, University of Toronto Mississauga Campus, Terrence Donnelly Health Sciences Complex, Room 354, 3359 Mississauga Rd, Mississauga, ON L5L 1C6 Canada; 3 Department of Family Medicine, McGill University, 5858, chemin de la Côte-des-Neiges, 3rd floor, Montreal, QC H3S 1Z1 Canada; 4 Epidemiology Division, University of Toronto, Dalla Lana School of Public Health, Toronto, ON Canada; 5 Leadership Sinai Centre for Diabetes, Mount Sinai Hospital, Toronto, ON Canada

**Keywords:** Indigenous Health, Infant feeding, Breastfeeding, Qualitative, Maternal and child health, Nutrition, Scoping review

## Abstract

**Background:**

Although exclusive breastfeeding is recommended for the first six months of life, research suggests that breastfeeding initiation rates and duration among Indigenous communities differ from this recommendation. Qualitative studies point to a variety of factors influencing infant feeding decisions; however, there has been no collective review of this literature published to date. Therefore, the objective of this scoping review was to identify and summarize the qualitative literature regarding Indigenous infant feeding experiences within Canada, the United States, Australia, and Aotearoa.

**Methods:**

Using the Preferred Reporting Items for Systematic Reviews and Meta-Analyses- Scoping Reviews and the Joanna Briggs Institute Guidelines, in October 2020, Medline, Embase, CINAHL, PsycINFO, and Scopus were searched for relevant papers focusing on Indigenous infant feeding experiences. Screening and full-text review was completed by two independent reviewers. A grey literature search was also conducted using country-specific Google searches and targeted website searching. The protocol is registered with the Open Science Framework and published in BMJ Open.

**Results:**

Forty-six papers from the five databases and grey literature searches were included in the final review and extraction. There were 18 papers from Canada, 11 papers in the US, 9 studies in Australia and 8 studies conducted in Aotearoa. We identified the following themes describing infant feeding experiences through qualitative analysis: *colonization, culture and traditionality, social perceptions, family, professional influences, environment, cultural safety, survivance, establishing breastfeeding, autonomy, infant feeding knowledge*, and *milk substitutes*, with *family* and *culture* having the most influence on infant feeding experiences based on frequency of themes.

**Conclusions:**

This review highlights key influencers of Indigenous caregivers’ infant feeding experiences, which are often situated within complex social and environmental contexts with the role of family and culture as essential in supporting caregivers. There is a need for long-term follow-up studies that partner with communities to support sustainable policy and program changes that support infant and maternal health.

**Supplementary Information:**

The online version contains supplementary material available at 10.1186/s12889-024-19060-1.

## Introduction

Nutritional status is a key aspect of infant health with recommendations for exclusive breastfeeding for the first six months of life, which can also influence and be influenced by maternal health and wellbeing [1, 2]. Breastfeeding has several benefits for the health and development of infants, including a reduced risk of ear and respiratory infections, obesity, asthma, skin conditions, childhood leukemia, and gastroenteritis [3–5]. It also supports bonding between the child and parent with improved intimacy [3]. Additionally, breastfeeding has several maternal physical and mental health benefits, including a reduced risk of breast and ovarian cancer, depression, and type 2 diabetes due to immunoprotective antibodies in breastmilk [3]. The World Health Organization (WHO) recommends exclusive breastfeeding for the first 6 months of life and initiation within the first hour after birth; however, less than half of infants 0–6 months old are exclusively breastfed worldwide [6]. Many countries are not meeting the WHO recommendations, with notable differences between low, middle, and high-income countries [2]. Differences in breastfeeding initiation rates and duration have been observed between Indigenous and non-Indigenous groups, with 6–10% lower breastfeeding initiation rates and shorter duration for Indigenous peoples [7–9].

Despite the many benefits of breastfeeding, bottle feeding with milk substitutes is a common form of infant nutrition and its common usage is related to a multi-dimensional set of factors influencing infant feeding decision-making. Breastfeeding is considered a traditional practice within many Indigenous cultures; however, disruptions to traditional lifeways through colonization have influenced intergenerational knowledge sharing, particularly within high-income, settler states like Canada, the US, Australia, and Aotearoa (New Zealand) [10]. Rollins et al. [1] summarize factors that influence the global breastfeeding environment including the sociocultural and market contexts, the healthcare system and services, family and community settings, employment, and individual determinants like the mother and infant attributes. However, these core breastfeeding environments for general populations overlook key considerations for Indigenous communities given the unique historical, cultural, and socio-economic contexts specific to Indigenous groups [11].

Many studies to date have focused on quantitative infant feeding data, incorporating structured questionnaires that have provided some insight into breastfeeding barriers and enablers for Indigenous caregivers [7, 12–14]. However, these studies are informed by specific research questions and do not capture important nuances that caregivers experience related to infant feeding. Qualitative research can enhance our understanding of phenomena by providing flexible means for participants to engage in the research topic of interest without the constraints of structured instruments, and can even transform the research by highlighting community needs [15, 16]. Qualitative research can also have synergy with Indigenous methodologies, supporting the use of qualitative research with Indigenous communities [17]. Given the value of qualitative inquiry and breastfeeding as traditional practice for many Indigenous cultures, disrupted by colonial influences and the burden of conditions that breastfeeding has been shown to mitigate [3, 5, 10, 11, 16, 17], it is imperative that we consider Indigenous caregiver infant feeding experiences and perspectives to understand what needs exist as defined by communities and caregivers. Therefore, the overall aim of this scoping review was to identify and summarize the qualitative literature on infant nutrition experiences to inform needs as expressed qualitatively by Indigenous caregivers in Canada, the US, Australia, and Aotearoa. These regions are included given the shared colonial influences on Indigenous peoples with overlapping outcomes on health [10, 18]. This review will also assess the qualitative methodologies used to understand what can be learned to inform Indigenous infant feeding services, policies, and research gaps.

## Methods

### Protocol and registration

This scoping review adheres to guidelines from Tricco and colleagues’ [19] *Preferred Reporting Items for Systematic Reviews and Meta-Analyses* (*PRISMA) extension for scoping reviews*, the Joanna Briggs Institute’s *Reviewer’s Manual Chap. 11* [20], as well as Arksey & O’Malley’s [21] foundational article on scoping studies. The protocol for the review is registered with the Open Science Framework (10.17605/OSF.IO/J8ZW2) and published with BMJ Open [22].

### Eligibility criteria

Works included in this review must have focused on Indigenous populations in Canada, the United States, Australia, and/or Aotearoa. These four countries share commonalities in that they are colonial countries in which Indigenous peoples face inequitable health outcomes [10, 18, 23]. The topic of interest for this review was caregivers’ experiences of infant feeding within one or more of these regions. “Caregivers” refer to individuals in the infants’ immediate familial and social circles who are directly responsible for the regular care of the infant. A broad definition of those involved in caregiving was used, recognizing that within many Indigenous communities, traditional adoption practices occur, or biological parents may not be the primary caregivers in part related to complex socio-ecological challenges. The experiences of healthcare professionals were not included as they were not considered “caregivers” by this definition. Works that discussed breastfeeding, as well as alternative forms of infant feeding, such as formula and cow’s milk, were included. Works that only focused on the introduction of solid foods were excluded. To capture caregivers’ *experiences* of infant feeding, qualitative and mixed-method studies that discussed experiences, perspectives, and/or practices as described by caregivers were included. Studies that used exclusively quantitative methods or that only described an outsider perspective (e.g. health professional) were excluded. Peer-reviewed journal articles and grey literature were included if they met the above criteria, were published in the English language, and were published after 1969 [22].

Various types of grey literature such as government documents, dissertations, and research reports by academic and non-academic institutions, including Indigenous organizations, were included. Media reports (including videos, news, and blogs) were excluded from the grey literature as they did not follow a research design with results that could be considered alongside the studies included in the review, hindering our ability to compare and critically analyze the results. Similarly, publications that consisted of only an abstract were excluded from both grey and database publications during full-text review as not enough information was present for analysis.

### Information sources

The search strategy was created with guidance from a research librarian at the Gerstein Science Information Centre, University of Toronto. The complete search strategy can be found as supplementary material in our published protocol [22]. Search terms primarily included broad terminology for Indigenous peoples (e.g. Native American) rather than specific Nation names (e.g. Ojibwe) as this would have significantly extended the search term list while not resulting in additional sources given how sources are indexed within Library systems. A database and grey literature search were conducted for this scoping review, completed independently from one another until final data extraction when the data were combined for analysis. For both searches, the reviewers followed a step-by-step process of title and abstract screening, followed by full-text screening, and then data extraction.

The database search planning and calibration occurred in August and September of 2020, and all data were exported in English on October 20, 21, and 22 of 2020. Exportation occurred over three days given feasibility of exporting the high number of citations and time capacity of the reviewers. A total of 16734 relevant sources available in the following databases were included: Medline, Embase, CINAHL, PsycINFO, and Scopus. These databases were selected to ensure a broad range of research given the multidisciplinary nature of research on this topic. The grey literature search consisted of a targeted search of a variety of Indigenous focused websites specific to the four countries and a thorough Google search with each of the country-specific Google versions (Google.com.au, Google.co.nz, Google.ca, and Google.com) where the first 10 pages of results were reviewed (Supplementary File 1). Lastly, Indigenous Studies Portal (I-Portal) was searched as part of the grey literature as this database uses a different indexing system than other research databases. The Canadian Agency for Drugs and Technologies in Health (CADTH)’s “Grey Matters” checklist [24] was used in the planning and tracking of grey literature searches and findings.

The results of the database search including 16734 citations were uploaded to Covidence (Veritas Health Innovation Ltd., Melbourne, Australia), a data management platform for systematic and scoping reviews, where 3928 duplicates were automatically removed. The 284 results of the grey literature search were recorded on Google Sheets (Alphabet Inc. California, USA) and 146 duplicates were manually removed by the reviewers. Due to the large number of results retrieved in the database and grey literature search, a hand-search of reference lists was not conducted.

A list of key words developed by HM were searched on each site and can be found in Supplementary File 1. The grey literature search was completed by HM, CC, and HS with all reviewers assigned to search a Country-specific Google database for one of the included countries. Using a template created by Stapleton [25] at the University of Waterloo based on methods described by Godin et al. [26], the reviewers kept track of which search terms were searched on the websites, the number of results retrieved, and the number of items screened and saved for further full-text analysis. If a website did not have a search bar, relevant tabs were examined for research, resources, and other publications. I-Portal was originally searched on August 15th, 2021 (yielding 10 results), however the search was revised to remove Indigenous search terms as the database was already Indigenous-specific. The search was repeated on August 18th, 2021, and yielded 77 additional results. The grey literature search was completed between May 25, 2021 – August 18, 2021. No search limitations or filters were used for the grey literature search or the database search.

The database abstract screening was initially completed by HM and CC starting in October 2020. They were then joined by HS and CL in February 2021. To ensure all reviewers had a shared understanding of the eligibility criteria, two search results were screened together and each reviewer discussed their reasoning for inclusion or exclusion. HM also hosted an introductory meeting to review the screening process using Covidence Software [27] in detail. All 12806 database results were saved in Covidence [27].

Abstract and full-text screening was completed in Covidence by two independent reviewers. Any conflicts at the screening stage were resolved by AH after all the results had been screened by two reviewers. Full-text screening was completed by HM, AH, and CC, and when conflicts arose, the reviewers met to discuss the difference in opinion until a consensus was reached. A third reviewer joined to offer impartial opinions for full-text conflicts.

Grey literature results were not imported to Covidence. Instead, the team used Google Sheets to organize the publications. Similar to the database review process, each study was screened by two independent reviewers and conflicts were resolved by a third party and discussed for consensus. Full-text review of the grey literature was completed by HM, AH, CC, and HS.

### Data extraction and analysis

HM compiled a list of variables to extract (Supplementary File 2), and the data extraction was completed by HM, AH, and CC in Covidence for database results and Google Sheets for the grey literature. The extraction template was reviewed and tested by all three reviewers using the same two articles. Discussion about any areas of confusion followed by minor edits to the data extraction template were completed prior to extraction.

Only one reviewer extracted data from most publications, however in circumstances where an article was complex or data extraction was not clear given the format of the article, two reviewers extracted data from the publication. An additional subset of five publications were also randomly double-reviewed by HM to ensure consistency in data extraction. There were an additional two articles that were excluded at this step after review and discussion by AH and HM.

Review findings using the extraction template (supplementary file 2) were exported into Microsoft Excel (Microsoft Corporation, Washington, USA) and reviewed by HM. HM compiled all data and completed summary figures for variables of interest. The primary analysis consisted of a qualitative review of the included papers’ results and recommendations using a thematic synthesis informed by grounded theory and meta-ethnography, where the included papers are synthesized together, and interpreted using descriptive and analytical themes [28]. Similar to grounded theory, this process was inductive and identifies themes through comparisons. HM reviewed all extracted data from the excel files, coding for overlapping themes and taking notes throughout. The full-text of the extracted papers were then revisited to identify overall concepts, followed by descriptive themes. Categorization of descriptive themes was completed based on the results and interpretations of included papers. Descriptive themes were refined through additional comparisons between papers. The same analytical process was used for both database and grey literature results, and final analysis involved the integration of themes from the database and grey literature papers. Supplementary file 3 provides a summary table of the included papers in this scoping review.

### Characteristics of included articles

**Fig. 1:**
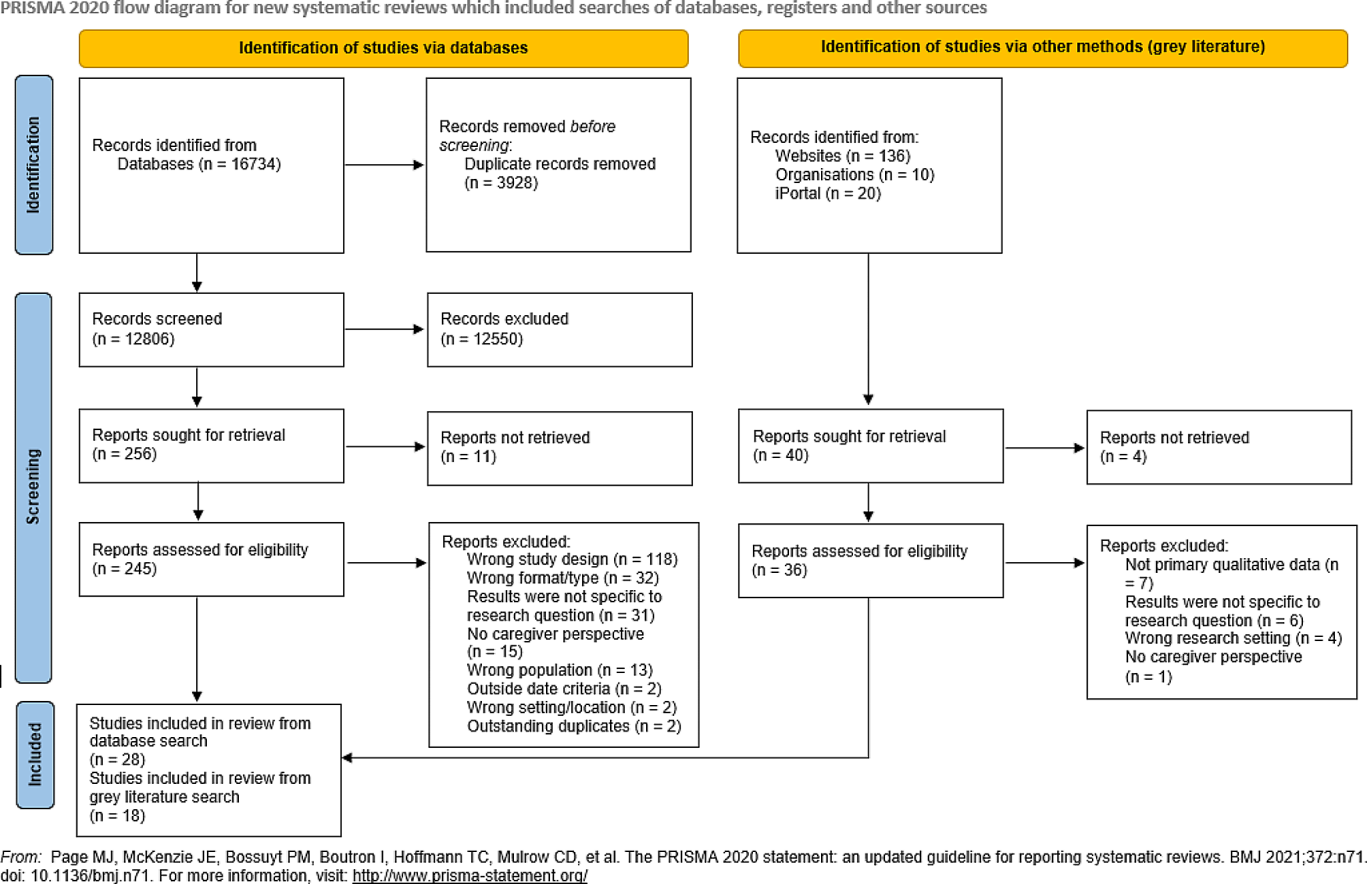
PRISMA flow diagram for studies identified, screened, and included in this review from both database and grey literature searches. Note that records not retrived are those in which the full-text was not accessible. This diagram was created from the PRISMA 2020 statement [29]

**Fig. 2:**
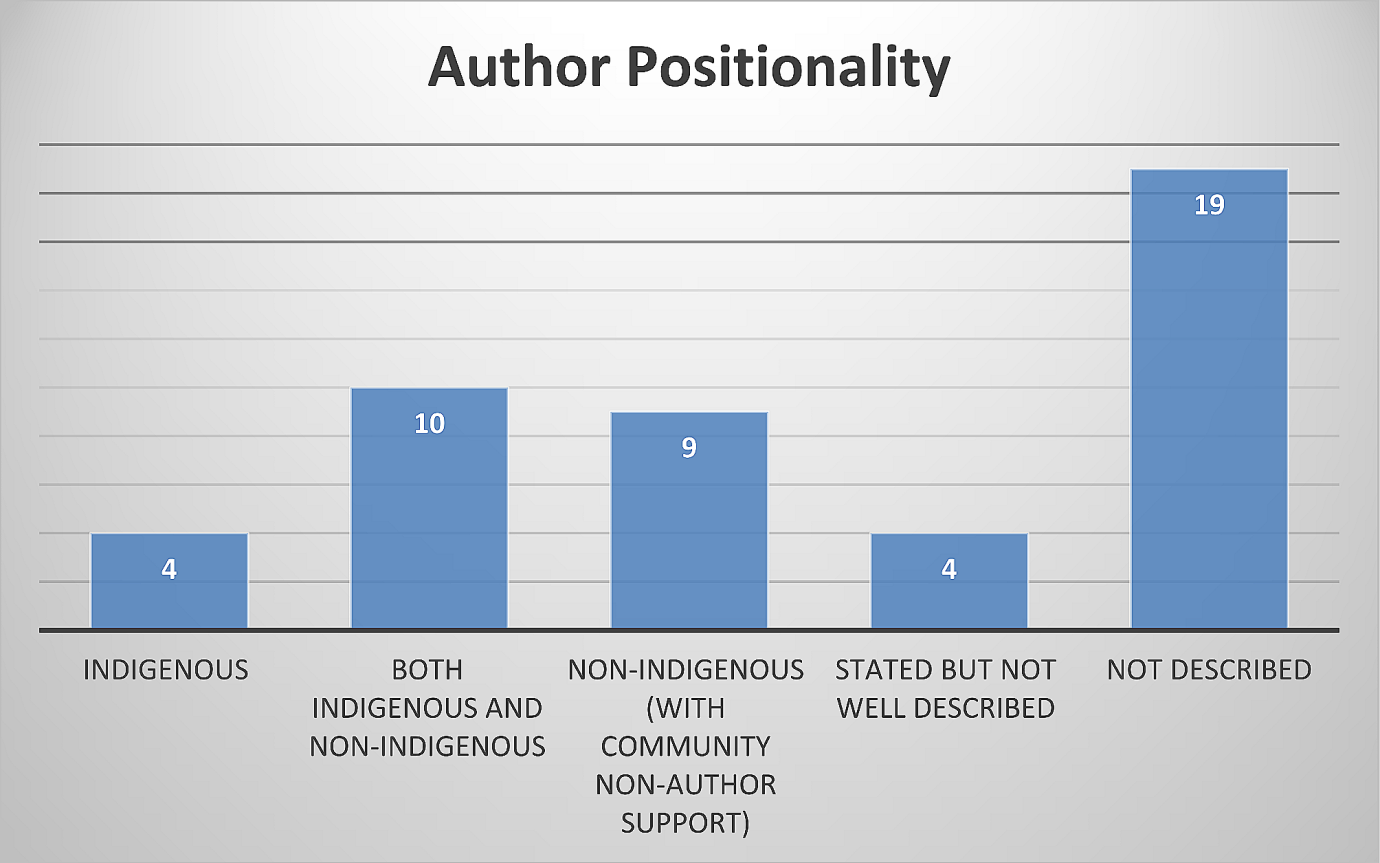
Author positionality as described in the retained papers

**Fig. 3:**
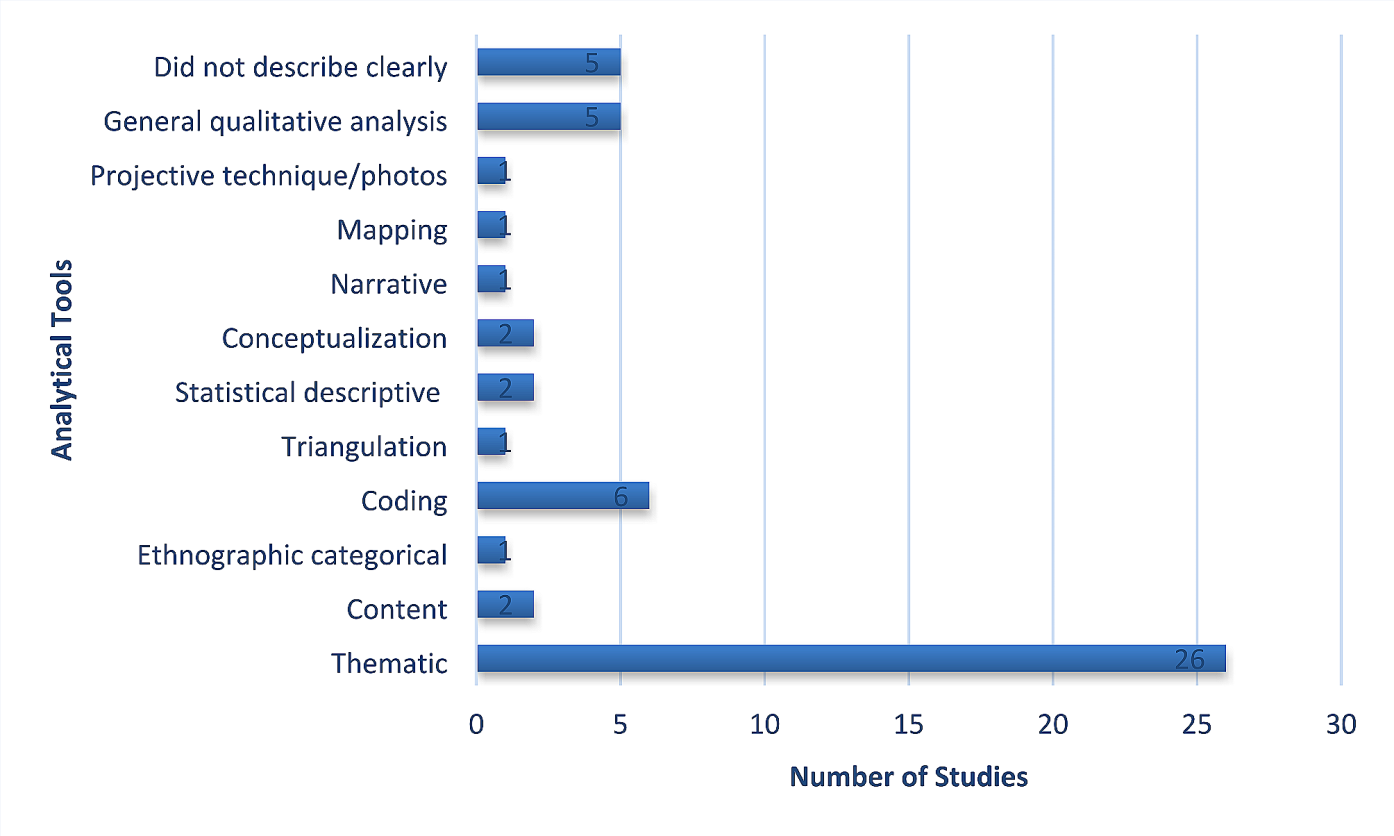
Summary of analytic tools used in the retained studies

**Fig. 4:**
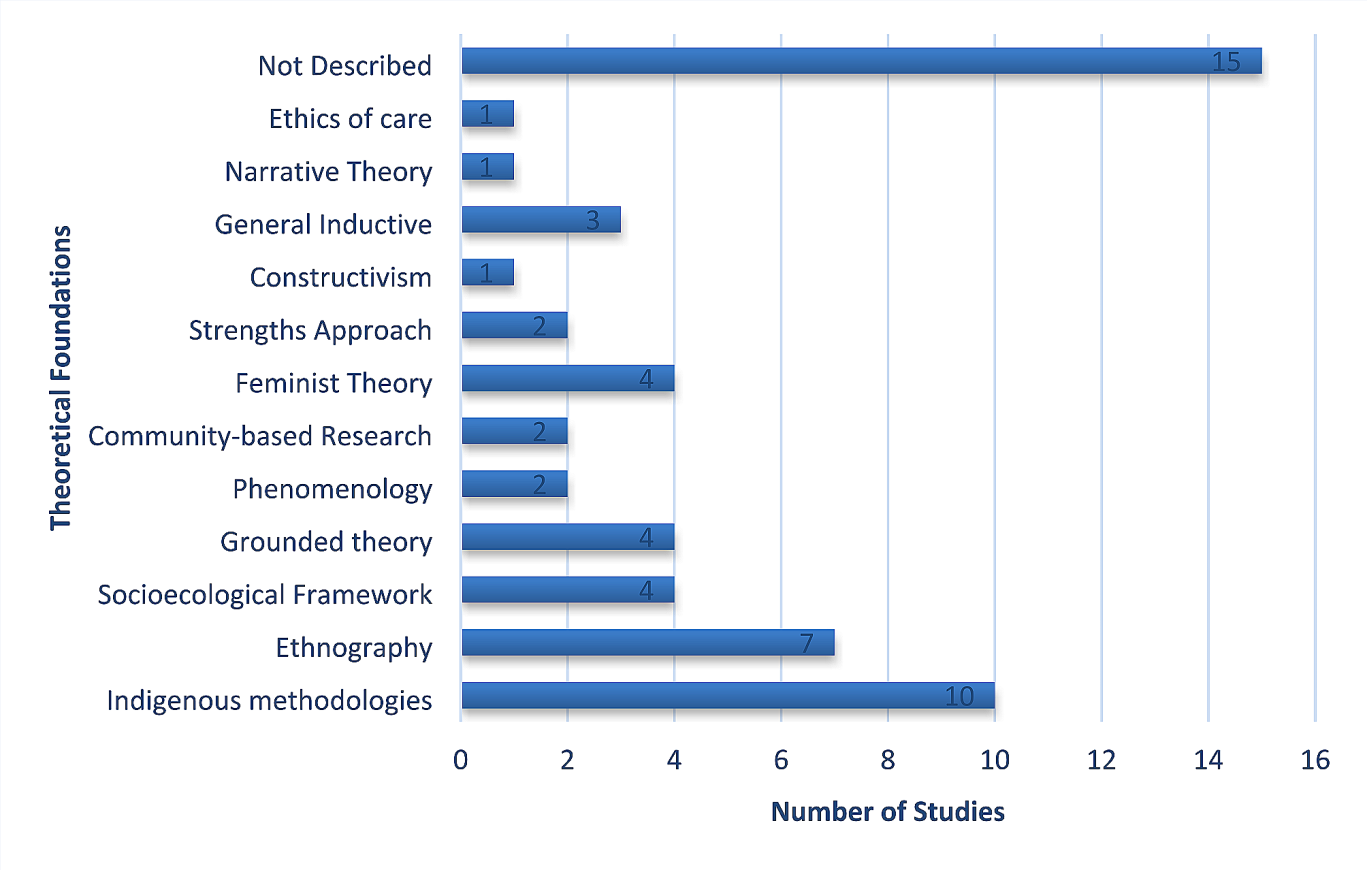
Summary of theoretical foundations informing the retained studies’ methodologies

Of the final sample of 46 articles from which data was extracted (Fig. 1), there were studies from each of the four countries, with the most studies (39%) published from Canada. In addition, this qualitative literature on infant feeding included several Indigenous groups within the four countries. The studies retained in this review included authors who identified as either Indigenous or non-Indigenous, and several did not mention positionality (Fig. 2). 13% more grey literature studies discussed positionality and had Indigenous sole authorship compared to the database papers. Regarding methodologies utilized, several described Indigenous methodologies and used thematic analysis as an analytic tool (Figs. 3 and 4). However, a third of the studies did not describe their theoretical foundations for the qualitative inquiry. Over 60% of the studies were published in the fields of public health and/or nursing as per the authors stated fields of study and/or the Journal’s field, and although there were studies published from 1984 to 2019, 50% of the retained papers were published after 2010.

## Results

**Fig. 5:**
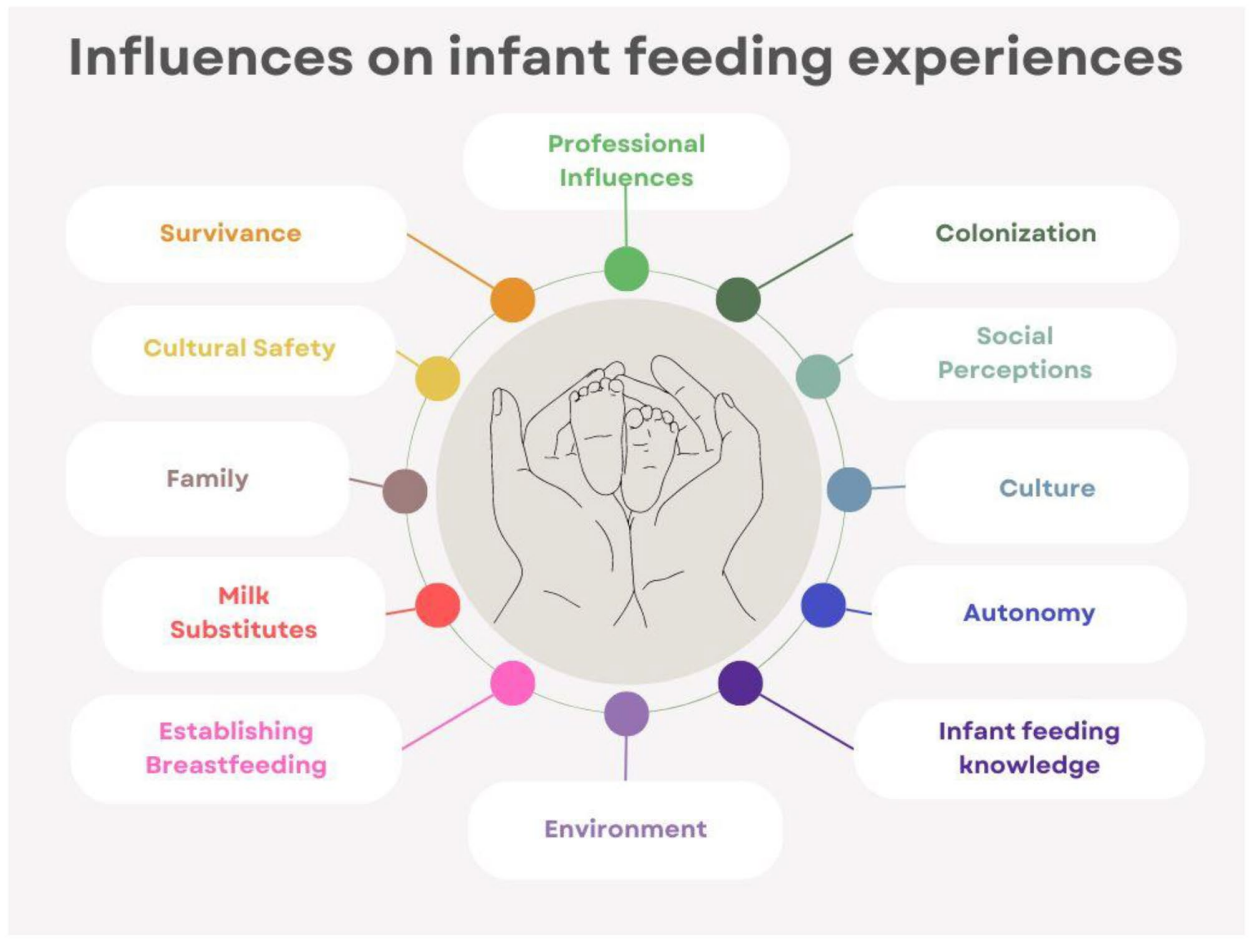
Scoping review research model of themes The twelve final themes are shown as the main influences on infant feeding experiences. The themes are arranged in a circular pattern with the infant and caregiver represented at the centre, emphasizing the connection between all of the themes

Analysis revealed a variety of important themes that aligned with Indigenous and public health perspectives on health, including the socioecological model. There were twelve final overarching themes including *colonization, social perceptions, family, professional influences, culture and traditionality*, *environment (i.e. built environment)*, *autonomy, survivance, infant feeding knowledge, cultural safety*, *milk substitutes*, and *establishing breastfeeding* with evidence of connections among these themes. These themes are shown in Fig. 5 in a circular pattern where the themes intersect with the infant and caregiver represented at the centre. This model is conceptually aligned with that of Dodgson et al. [30], who considered the “contextual influences within the social structures of family and community, Ojibwe culture, and mainstream culture.”

### Theme one: colonization

**Fig. 6:**
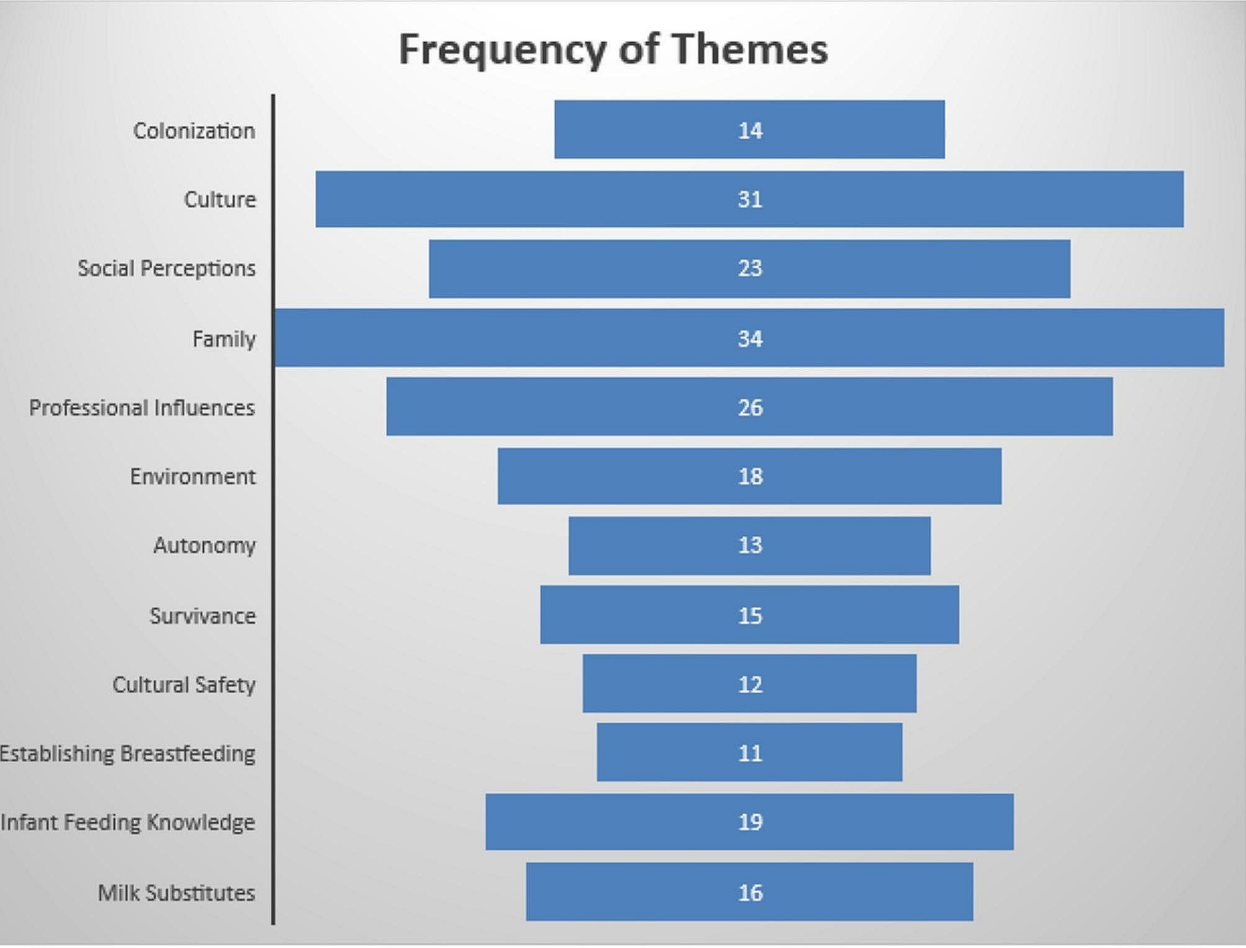
Frequency of identified themes in the database papers and the grey literature

There were 14 papers that discussed *colonization* of Indigenous peoples as a key factor influencing infant feeding decisions and experiences (Fig. 6) [30–43]. Colonization has meant the dispossession of land and limited access to culturally safe healthcare, malnutrition, and loss of language through residential schools, loss of culture and traditional knowledge through assimilation and separation of families, disrupting breastfeeding practices and limiting income for infant formula. Eni et al. [36] described the policies leading to evacuation from communities to tertiary-care hospitals for birthing as the medicalization of birthing practices, which creates various challenges for First Nations women in Canada. One participant also shared about the impacts of intergenerational trauma related to colonization on breastfeeding, ‘‘You can’t teach about breastfeeding technique and think things will change. It’s the spirit that’s been affected, our experience with trauma. Our women need to relearn how to bond with their children.’’.

A qualitative study with Aboriginal Australian first-time mothers noted the disruptions to breastfeeding practices over time, providing a historical chart detailing how infant feeding practices changed as a result of colonial influences [38]. Brittany Luby [39] described how hydroelectric flooding from 1900 to 1975 in Northwestern Ontario reduced breastfeeding practices for Anishinabek mothers and their infants. Although not all studies specifically discussed history and colonization, those that considered the broader historical context highlighted how important this issue is in understanding the factors that lead to infant feeding decisions, particularly those that do not align with breastfeeding as a traditional feeding practice.

### Theme two: culture and traditionality

*Culture*, including traditionality, was the second most described theme throughout all papers, identified both directly and indirectly in 31 papers (Fig. 6) [30–32, 34, 35, 37–42, 44–63]. The Navajo Infant Feeding Project focused on cultural beliefs influencing infant feeding practices within three Navajo communities in the United States [48] and emphasized breastfeeding’s significance for nutritional, physical, and psychological health where mothers not only pass along physical health benefits, but also their wellbeing to their children. The Baby Teeth Talk Study in Cree communities in Northern Manitoba, Canada, has identified breastfeeding as a cultural intervention for the prevention of early childhood caries [52]. Several studies included a variety of generations in data collection, contributing to rich discussion of how breastfeeding rates and connection to traditionality has changed in some communities [48, 57, 64, 65]. For example, grandmothers living on the Fort Peck Reservation in Montana, US, were interviewed about their perspectives on infant feeding [65]. In one of the ethnographic studies, there was a specific focus on the Ojibwe culture relating to infant feeding practices from the perspective of mothers, professionals who were also community members, and Elders [35]. This study emphasized the holistic and collective worldview of the community, influencing women’s roles within the family and how teachings were passed on from generation to generation [35]. This was considered to be important in influencing effective and culturally safe breastfeeding promotion. Within the Northwest Territories, Canada, Moffitt and Dickinson [53] supported breastfeeding knowledge translation tools for Tłı̨chǫ women with one of the themes focused on factors that “pull to breastfeeding,” including breastfeeding as a traditional feeding method. In general, Indigenous communities described breastfeeding as a cultural practice; however, how this is supported and the traditional knowledge surrounding this practice may differ from community to community. Therefore, health providers must be aware of community-specific protocols and support these within programs and recommendations.

### Theme three: social perceptions

Societal influences are often considered alongside cultural perspectives of infant feeding; therefore, this theme was also commonly discussed in the papers retained in this scoping review (Fig. 6) [30, 32, 33, 36–38, 40, 42, 49, 50, 52, 54, 57–59, 61, 64, 66–71]. In New South Wales, Australia, Aboriginal mothers and key informants noted the need for “a safe place to feed,” including concerns about the social acceptability to breastfeed in public [32]. Broader social “norms” are also discussed as influencing maternal behavior [68], and respondents in some studies expressed concern about judgements from others [32, 36]. Tapera et al. [40] described concerns about social pressures and a lack of support with one grandparent sharing, “well here in New Zealand, I know we have a problem with this [breast-feeding], especially when mothers go out and they breast-feed their babies in public. There’s a lot of people that moan and groan about this.” Similarly, regarding social norms, a grandmother living in the US shared,

“a long time ago that, it [breastfeeding] was acceptable and nobody had any qualms about it but today, I mean you read continually about, people, mother’s tryin’ ta breastfeed and they’re being chased out a places or stores or people are rude about it […]. Society’s changed, you know, it’s […] society, has come to the point where it’s […] trying to tell us what’s the right way ta live what’s the right way ta raise our kids” [65].

Dodgson et al. [30] described how in an Ojibwe community in Minnesota, US, participants noted the dominant societal influences in contrast to community traditions, with women making an effort to engage in traditional practices. The sexualization of breasts in mainstream society sometimes influenced Indigenous mothers’ infant feeding experiences [36], although Ojibwe caregivers in Minnesota attributed shyness with breastfeeding to traditional value opposed to sexualization of breasts [30]. Eni et al. [36] included *sexual objectification of the feminine body* as a subtheme in their study, describing how this social perception damages maternal mental health, creating a barrier to breastfeeding. While shifting social norms is a significant challenge, breastfeeding supports can address concerns about the sexual objectification of breasts by creating safe spaces for parents to talk about the challenges and ensure that parents have access to mental health resources.

### Theme four: family

Dodgson et al. [30] described *family* as a pattern that influences breastfeeding intersecting with the social structures of the community, culture, and the broader society. There were 33 other papers that described the influence of *family* on infant feeding practices making this the most discussed theme (Fig. 6) [30–33, 36, 38–40, 43–51, 53–55, 57–61, 65–73]. Native American mothers living in six communities highlighted the importance of *family* as a key theme [47]. One mother shared, “For me, it’s my mom definitely [whose advice is most important] because she has had three kids and I lived with her or near her for all of my kids. So I’ve always gone to her first for advice.” This was echoed by many other participants with a paraprofessional adding, “family [advice is most important], because they are around their family most. And they always hear from their aunties, or from grandma, baby’s fussing, baby must be hungry, baby needs this and baby needs that.” The Baby Basket Program in Cape York, Australia identified that *empowering families* was the foundation of the program to ensure that mothers and their partners were equipped for the arrival of their babies [50]. Family often plays an integral role in supporting mothers in infant feeding practices. Bauer and Wright [45] note that even when mothers don’t have other supports or conditions in place to support breastfeeding, they may still choose to breastfeed if their family is supportive. However, when this support is lacking, mothers find it challenging to breastfeed [31, 36]. Some studies identified the significance of family in the study design, integrating family caregiver perspectives in data collection [64, 65]. Therefore, health programs and research studies should consider the role and experience of non-primary caregivers within family networks for infant and maternal health and nutrition.

### Theme five: professional influences

This theme represents the influence of formal systems including healthcare professionals, health and social programs, child services, and the legal system. In total, there were 26 papers that referenced professional influences on infant feeding experiences (Fig. 6) [30, 31, 33, 38, 41–43, 45, 47, 48, 50–52, 54, 58, 59, 61, 62, 66–73]. Some studies incorporate health workers as participants in data collection [47, 50, 65]. One health paraprofessional shares about some of the pressures experienced by mothers to formula feed, “sometimes hospitals and doctors want to push formula in bottles on moms [47].” One of the main themes in a study with Sioux and Assiniboine Nations in the US was the ‘*Overburdened Healthcare System’*, describing a lack of resources and infrastructure to support breastfeeding, including a subtheme of *mistrust* in the healthcare system due to previous negative experiences such as forced sterilization of Indigenous women [65]. However, some caregivers also expressed positive healthcare supports, “when I was at home, [clinic midwife] and [lactation consultant made home visits] … they encouraged me … And then it started getting a little bit better, but it was still a bit hard. Now he feeds pretty all right [73].” Professional influences on infant feeding are nuanced and may differ significantly within various contexts and individuals; therefore, tailored interventions are needed.

### Theme six: environment

This theme represents the external variables within the built environment that influence decision making including work, school, remoteness, and cost of formula. Eighteen papers addressed this theme [30, 31, 44–49, 51, 53, 58, 59, 66–68, 70–72]. Wright et al. [74] specifically considered the challenge of breastfeeding with maternal employment among the Navajo population in the US. In Bauer and Wright’s [45] study that explored infant feeding decision models, they identified that work and school are part of the decision-making process on whether to breastfeeding or to use formula, but even when these environmental challenges are present they can be further influenced by other factors, like *family*. For example, a mother may choose to breastfeed and use a breast pump to navigate work/school schedules, but family members may recommend that they can incorporate formula; decision-making is not only about the main caregiver’s desires but can involve various decision-makers.

### Theme seven: autonomy

This theme describes parents’ freedom to make infant feeding decisions that fit for them and their priorities. Maternal desire to breast- or bottle-feed was discussed in select papers in this review [45, 51]. In addition, other papers describe parents’ freedom to do activities outside of infant feeding in the early months of baby’s life with discussion of time required to breastfeed or prepare bottles for feeding [31, 58, 72, 74]. A key informant in a study with an Aboriginal community in Northern New South Wales, Australia, shares, “they want to breastfeed, but then it comes down to when they want to go out, or keep up with their man [32].” Some parents report that they experienced judgements from others or feel forced into making a specific decision on infant feeding method, highlighting a desire to have support and freedom to make their own decisions [36, 56].

### Theme eight: infant feeding knowledge

Several studies emphasize the importance of knowledge on infant feeding experiences, highlighting the value of infant feeding education, both within the overall healthcare system and from traditional teachings [30, 32, 35, 40, 42, 43, 47, 52, 57, 58, 62, 64, 66–72]. Within the theme of *addressing feeding challenges* in one study [66], a caregiver shared how knowledge helped her to work through a challenge,

“He did start fussing at about 6 weeks and that was kind of hard because I thought, ‘No, I have got this perfect now, and he has started to muck up’. But then I read, because I had those booklets and I read that sometimes they — at a certain point — they get a bit fussy and you just have to work through it. [Ml7]” [66].

Traditional breastfeeding knowledge is important for many communities; one Anishinaabe community knowledge keeper shared that “breast milk is a gift and a medicine a mother gives her child” [35]. This study also discusses feeding patterns as shared by Elders and traditional teachers. Traditional knowledge considers holistic perspectives of health where caregivers are also focused on the baby’s spiritual wellbeing [48, 56].

### Theme nine: milk substitutes

Bottle feeding (formula or canned milk) and solid foods are described in several papers as alternatives or complements to breastfeeding [31, 33, 34, 37, 39, 47–49, 51–53, 58, 66, 67, 74, 75]. In Neander and Morse’s [37] study with a Cree community in Alberta, Canada, bottle feedings were offered particularly when mothers felt that they were not producing adequate milk supply to meet the baby’s nutritional needs. Insufficient milk supply is echoed as a concern in several other papers resulting in complementary bottle feeding or weaning [48, 51, 56, 66, 67]. A Māori father shares,

“about the second week, baby just wanted more food. She (partner) would end her day and baby was just hungry. We had to [give her] the bottle and then she would be finally satisfied. It wasn’t that she made a choice. Baby was actually demanding more and more and she couldn’t produce it. (First-time father, mid 20’s) [56].”

This theme particularly overlaps with *autonomy* as parents balance infant feeding decisions with breastmilk supply, work, school, and other personal commitments.

### Theme ten: cultural safety

Indigenous caregivers interact with a variety of health services postnatally; however, there is a need to address cultural safety within the healthcare system. Twelve retained papers highlighted this theme either directly as one of their themes or as part of another theme (Fig. 6) [30, 31, 44, 47, 50, 64, 66, 67, 69, 71, 73, 74]. One health worker in Victoria, Australia, shared,

“I can’t say often enough or long enough, loud enough the ideal for children 0–8 is to have access to maternal and child health. You might say ‘oh yes, they’ve got access to mainstream and they’re culturally going to put up a few Indigenous prints in their rooms’ It’s not the same. Our families are telling us with their feet it’s not the same.”

Mothers expressed a desire for more traditional infant feeding knowledge within services and culturally relevant supports [47, 64]. A study that focused on a baby basket program to support families in a Murri (Local Australian Aboriginal Group) Way identified how important culturally safe language and relationships are for families,

“…the nurse is also learning what the best way is to approach a family and what the wording has to be, what the languaging is around things, what the traditional words are for Indigenous language and are appropriate for use in certain circumstances” [50].

### Theme eleven: survivance

Indigenous caregivers experience a variety of hardships; however, through resistance and survival, they practice cultural revitalization [76]. This theme is discussed in 15 papers and is often described through a lens of maternal mental health (Fig. 6) [30, 31, 33, 43, 53, 54, 57–59, 63, 64, 66, 68, 36, 74]. Some parents express feelings of guilt for the challenges they encounter, which can further contribute to negative emotions [58]. Maternal mental and emotional health can impact infant feeding experiences,

“…sometimes people’s psychological health, mental health is more of a risk factor, you know if you’re not sleeping and you’re bordering on depression and you’re not coping well and you can’t get the baby to latch and you’re constantly feeling like a failure and you can’t get out of that rut, is it worth it?…People have to decide that for themselves. (Key Informant #5)” [33].

A grandmother in the Northwest Territories of Canada noted the disembodiment caused by residential schools as expressed as a disconnection between physical experiences and relationships,

“You know in those days, I mean residential school. In those days, they never did talk about their body parts because I think they were too ashamed [of your body] to say to your kids. I never did hear it [breastfeeding] from my sisters or nobody in the family. They were so private (L151-156)” [57].

Traumatic experiences, like residential schools, can have a lasting impact on how caregivers navigate motherhood and infant feeding, and the support they receive from family members.

### Theme twelve: establishing breastfeeding

There are several practical challenges that mothers encounter while breastfeeding like pain, latching issues, and low milk supply, discussed in 11 of the studies (Fig. 6) [48, 51, 54, 56, 58, 61, 66, 68, 71, 72]. A mother shared,

“He wouldn’t latch on all the time, like, the nurses and stuff tried to help me but then it would be all frustrating…. He didn’t really know what to do. He tried and then they gave him formula. He really loved it. [MI5]” [66].

Although these challenges are most discussed at the beginning of breastfeeding, sometimes concerns arise when babies are older.

“Yeah it was 8 or 9 months after she was born. After a while there was too much pressure on me. She was getting up all through the night and she would eat and eat and eat and not get full…” [33].

Overall, many caregivers reported that breastfeeding is difficult; therefore, supports that consider the variety of challenges that can arise are needed.

### Study recommendations

The studies included in this review were published over three decades starting in 1984 until 2019 and were completed with various Indigenous communities in four countries. We anticipated that earlier work would demonstrate markedly different infant feeding recommendations than more recent research; however, this was not necessarily the case. For example, cultural safety is a more recent discussion within the health literature; however, although we see some discussion of this in more recent studies, studies in the 80’s and 90’s also highlight the importance of incorporating traditional teaching and consulting community members [37, 48]. Therefore, supporting Indigenous self-determination where health professionals provide culturally appropriate care is essential.

**Fig. 7:**
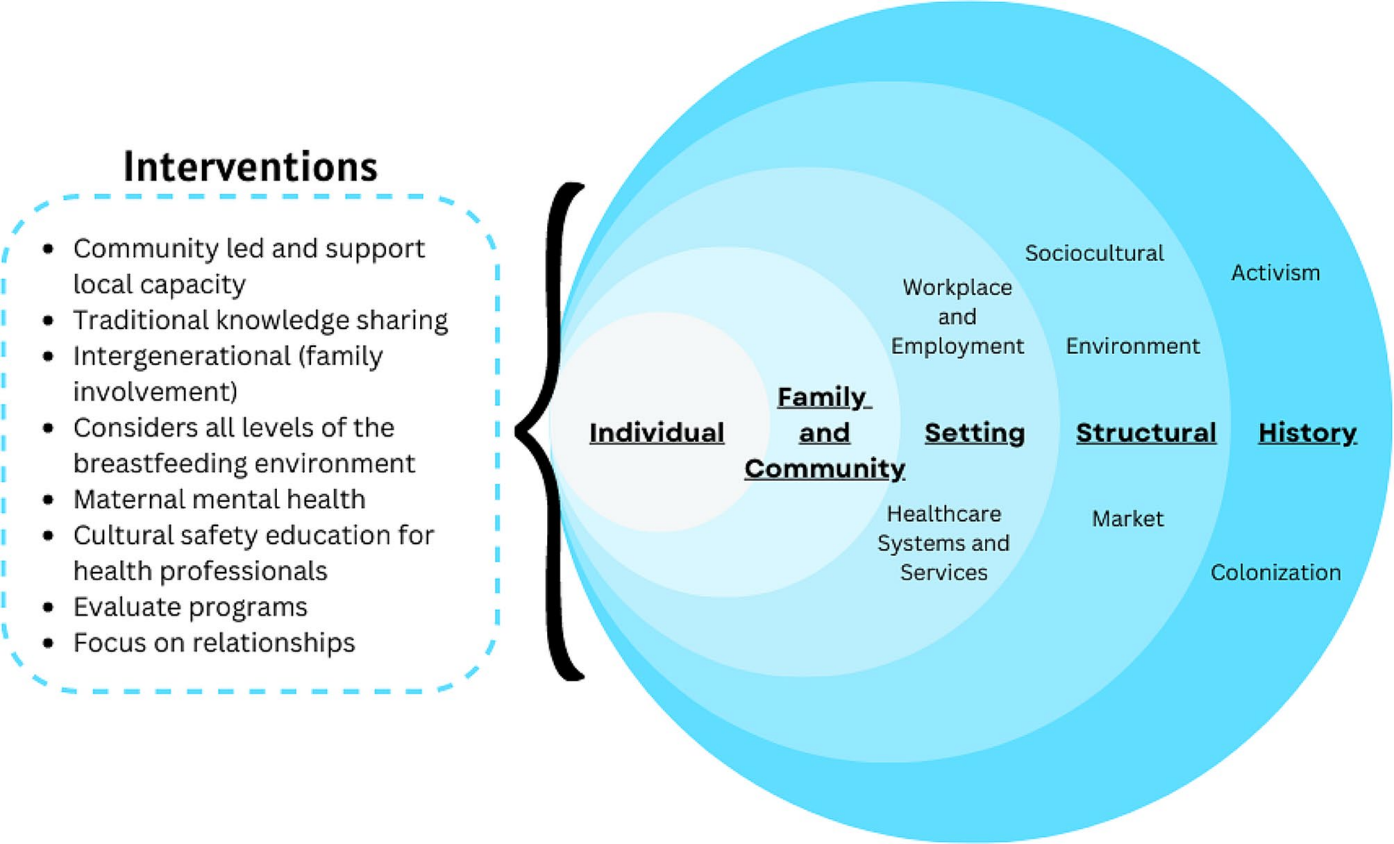
The components of Indigenous infant feeding environments informed by community-based interventions (Adapted from Rollins et al. 2016)

In addition to topics related to cultural safety, various studies highlight a need for community-driven and local knowledge to inform programs and policies related to infant nutrition [31, 47, 57, 64, 75]. Several studies also focus on infant feeding specific programs and behavioral changes in their recommendations [47, 50, 65]; however, many of these studies also highlight the need to expand beyond the individual’s role in decision making and address the broader social and environmental factors such as the workplace, healthcare infrastructure, social perceptions, among others, that influence infant feeding decisions. For example, Eni et al. [36] note that there are a complexity of factors resulting in various breastfeeding environments. These structural, social and cultural contexts are discussed throughout several of the grey literature texts as well [32, 33]. It is also important to note that in the most recently published database paper, maternal mental health is directly addressed in the recommendations and this is the only paper with this focus for next steps [65]. Interventions that target socio-ecological factors based on the included papers’ recommendations for infant feeding are summarized in Fig. 7.

## Discussion

This scoping review presents and summarizes the findings reporting Indigenous infant feeding experiences within the qualitative literature in Canada, the US, Australia, and Aotearoa. Twelve themes were identified which summarize the literature including *culture and traditionality*, *colonization, family, environment, social perceptions, professional influences, milk substitutes, breastfeeding initiation, cultural safety, survivance, infant feeding knowledge, and autonomy.* The most prevalent themes discussed by caregivers and researchers in the included papers were *family* and *culture/traditionality*. The frequency of these two themes highlight the significant impact of family and culture/traditionality on infant nutrition decision-making for Indigenous caregivers and overlaps with components of the socio-ecological model [77]. This focus on family and culture/traditionality also emphasizes the importance of familial relationships and a collective mentality within traditional life ways for many Indigenous communities in these regions on infant nutrition and care practices.

In their informative global breastfeeding paper, Rollins and colleagues’ [1] conceptualize the components that contribute to the breastfeeding environment at multiple levels, overlapping with the social determinants of health. In this review, we observed that caregivers report similar components of the breastfeeding environment; however, these components seem to be described collectively, rather than as separate contexts. This is evident in the recommendations proposed by authors with a large focus on local and community-specific leadership, multidisciplinary interventions, and cultural safety in response to historical traumas, particularly within the healthcare system (Fig. 7). This aligns with Indigenous epistemology with an emphasis on the collective and interconnectedness of all things where power is manifested together, not over one another, and is based in local land-based knowledge [78, 79].

A primary recommendation echoed within many of these studies was the need for community engagement in program and policy development [34, 47, 50, 64]. This may need to be expanded upon to support Indigenous self-determination of policy and programs related to infant feeding where community members are not only engaged but leading the way forward in maternal and infant health. It is important to note that there have been changes over time in how these recommendations and perspectives are discussed and the role of the health professional, particularly related to cultural safety. For example, although similar concepts are discussed in Neander and Morse’s paper published in 1989, ‘cultural safety’ is not used as the terminology, which has been expanded upon in recent years by Indigenous and non-Indigenous scholars [37, 80, 81].

Related to this focus on health professionals and cultural safety, it’s important to distinguish that in many of the positive experiences expressed by participants in the studies, these interactions seemed to be primarily with professionals interacting closely with families. For example, midwives, who make home visits, were often included as part of positive experiences. In the literature, there is an emphasis on including practitioners who can build strong relationships with families through home visits and regular community engagement in routine services, which supports cultural safety within the healthcare system [82, 83]. Health professional regulatory bodies should consider implementing practice competencies that support professionals to build and navigate strong and ethical relationships with clients/patients. Similarly, healthcare settings that serve Indigenous peoples should consider processes and therefore, facility infrastructures that enable close family-client-professional interactions. An example of this implementation with positive client experiences is the Toronto Birthing Centre, which uses an Indigenous framework and has birthing rooms with space for family [84].

The studies in this review are written within various fields of research; therefore, there were differences in methodological reporting. Future qualitative work should be thorough in reporting theoretical foundations to provide clarity of how the analyses and overall projects are approached (Fig. 4) [85]. Given the limited studies that report author/researcher positionality (Fig. 2), this may be an important addition in forthcoming work as a means of respecting Indigenous and qualitative literature conventions where we recognize that positionality influences ontological origins [86]. We challenge the academy to recognize that Indigenous and local knowledges are required within Indigenous health research and dissemination practices, while acknowledging our own limitation in this review of a single country authorship team.

This systematic scoping review utilized a rigorous search strategy that limited the possibility of missing relevant publications; however, it was time intensive. PRISMA-ScR guidelines were followed with two independent reviewers at each stage, enabling reproducibility of this review. The inclusion of the grey literature is a strength in this study as it captured important papers that were not published in peer-reviewed journals, often from Indigenous authors and communities (many of which were graduate dissertations), which was a priority in this review. A possible limitation is the exclusion of work that only discussed the introduction to solid foods; it is possible that this excluded an important conversation about the differences of introducing solids, like traditional foods from an Indigenous group’s perspective. In addition, the topic of this review is multidisciplinary; therefore, it is possible that although effort was made to include a broad range of research field databases in the search, relevant sources may have been missed.

In conclusion, this scoping review highlights important considerations for infant feeding environments within Indigenous communities with a focus on family and culture. Based on caregiver experiences, Indigenous breastfeeding supports must be community led with a focus on local capacity and traditional teachings. An emphasis on an intergenerational perspective that considers structural and systems approaches including cultural safety within healthcare, addressing maternal mental health, and consideration of sustainability over time is encouraged. Future work should focus on these key areas through strength-based research approaches, grounded in strong relationships and long-term follow-up.

### Electronic supplementary material

Below is the link to the electronic supplementary material.

Supplementary Material 1

Supplementary Material 2

Supplementary Material 3

Supplementary Material 4

## Data Availability

All data generated or analysed during this study are available from the corresponding author on reasonable request.
