# Supplementary material for: Infant feeding experiences among Indigenous communities in Canada, the United States, Australia, and Aotearoa: a scoping review of the qualitative literature
Source: BMC Public Health. 2024 Jun 13;24:1583. doi: 10.1186/s12889-024-19060-1 (PMC11170823; doi:10.1186/s12889-024-19060-1)
Supplement: Supplementary file 1 — Supplementary Material 1 [file 12889_2024_19060_MOESM1_ESM.docx]

| Search Engine | Search terms |
| --- | --- |
| Google.ca | 1. Indigenous infant feeding experiences 2. Indigenous breastfeeding experiences 3. Indigenous early life nutrition 4. Aboriginal infant feeding experiences 5. Aboriginal breastfeeding experiences 6. Aboriginal early life nutrition 7. First Nations infant feeding experiences 8. First Nations breastfeeding experiences 9. First Nations early life nutrition 10. Inuit infant feeding experiences 11. Inuit breastfeeding experiences 12. Inuit early life nutrition 13. Métis infant feeding experiences 14. Métis breastfeeding experiences 15. Métis early life nutrition |
| Google.au | 1. Indigenous infant feeding experiences 2. Indigenous breastfeeding experiences 3. Indigenous early life nutrition 4. Aboriginal infant feeding experiences 5. Aboriginal breastfeeding experiences 6. Aboriginal early life nutrition 7. Torres strait islander infant feeding experiences 8. Torres strait islander breastfeeding experiences 9. Torres strait islander early life nutrition |
| Google.nz | 1. Indigenous infant feeding experiences 2. Indigenous breastfeeding experiences 3. Indigenous early life nutrition 4. Māori infant feeding experiences 5. Māori breastfeeding experiences 6. Māori early life nutrition |
| Google.com | 1. Indigenous infant feeding experiences 2. Indigenous breastfeeding experiences 3. Indigenous early life nutrition 4. Native American infant feeding experiences 5. Native American breastfeeding experiences 6. Native American early life nutrition 7. American Indian infant feeding experiences 8. American Indian breastfeeding experiences 9. American Indian early life nutrition 10. Alaska Native infant feeding experiences 11. Alaska Native breastfeeding experiences 12. Alaska Native early life nutrition |
