# Supplementary material for: Infant feeding experiences among Indigenous communities in Canada, the United States, Australia, and Aotearoa: a scoping review of the qualitative literature
Source: BMC Public Health. 2024 Jun 13;24:1583. doi: 10.1186/s12889-024-19060-1 (PMC11170823; doi:10.1186/s12889-024-19060-1)
Supplement: Supplementary file 2 — Supplementary Material 2 [file 12889_2024_19060_MOESM2_ESM.docx]

**General information**

**Title**

Title of paper / abstract / report that data are extracted from

**Date of publication**

**Lead author name**

**Country in which the study conducted**

1. Canada
2. United States
3. New Zealand
4. Australia

**Name of Indigenous group(s) (ie. Haudenosaunee)**

indicate urban community if applicable and if they do not describe groups

**Name of Indigenous community (ie. Sandy Lake)**

if an urban community, name the city or organization

**Notes**

**Field of Research (ie. Nutrition, Anthropology, Nursing)**

**Characteristics of included studies**

**Methods**

**Aim of study**

**Study design**

1. Qualitative Research
2. Mixed Methods
3. Survey Data
4. Other

**Year(s) of research**

**Study funding sources**

**Method of recruitment of participants**

1. Phone
2. Mail
3. Clinic patients
4. Voluntary
5. Other

**Total number of participants**

**Positionality of researchers**

1. Indigenous
2. non-Indigenous
3. both Indigenous and non-Indigenous
4. Did not describe

**Elaborate on positionality of researchers and/or participants (if applicable)**

**Who participated?**

1. Mothers
2. Fathers
3. Grandparents
4. Health workers
5. All of the above
6. Other

**Method for data collection**

1. Interviews
2. Sharing or talking circles
3. Focus groups
4. Surveys
5. Observation
6. Art/visual techniques
7. Other

**Theoretical foundations**

1. Grounded theory
2. Indigenous theories/methodologies
3. Community-based participatory research (CBPR)
4. Narrative theory
5. did not describe
6. Other

**Analytical Tools**

1. thematic analysis
2. participatory analysis
3. content analysis
4. coding
5. did not describe
6. Other

**Results**

**What was the primary outcome/finding?**

**Did they describe findings as key themes?**

1. Yes
2. No

**List main themes if applicable**

**How are results described? (discussion of themes, figures etc.)**

**Are the results specific to infant feeding (yes) or only part of the outcomes? (No- eg. dental care where breastfeeding is a theme)**

1. Yes
2. No

**Notes on the focus of the paper (if applicable)**

**What are the barriers to breastfeeding? (if described)**

**What are the barriers to infant feeding? (if applicable)**

more general, if not described above

**What are the enablers/supports available for infant feeding?**

if applicable

**List study limitations as described by authors**

if applicable

**What recommendations are made for infant feeding (supports)?**

**Other important notes/results**
