# Supplementary material for: Infant feeding experiences among Indigenous communities in Canada, the United States, Australia, and Aotearoa: a scoping review of the qualitative literature
Source: BMC Public Health. 2024 Jun 13;24:1583. doi: 10.1186/s12889-024-19060-1 (PMC11170823; doi:10.1186/s12889-024-19060-1)
Supplement: Supplementary file 3 — Supplementary Material 3 [file 12889_2024_19060_MOESM3_ESM.docx]

| **Study Reference** | **Country in which the study was conducted** | **Name of Indigenous group(s)/community** | **Total number of participants** | **Method for data collection** | **What was the primary outcome/finding and if applicable, what were the study themes?** |
| --- | --- | --- | --- | --- | --- |
| Wright et al. 1993 | United States | Navajo; Kayenta, AZ and Shiprock and Gallup, NM | Phase 1 (ethnographic interviews): 35. Phase 2 (survey): 250. | Phase 1: ethnographic interviews; Phase 2: structured, open-ended questionnaire | 1. Work was a barrier to starting / maintaining breastfeeding;  2. Other (non-work) reasons why women chose to change their feeding practices (free time to do other things; fear of weight gain due to eating more during breastfeeding; insufficient milk) |
| Jones et al., 2017 | Aotearoa | Māori; Aotearoa | 58 for quantitative, 10 for qualitative | Online survey for quantitative, face-to-face interviews for qualitative | Being held and breast or bottle fed to sleep were the most practiced techniques by Māori parents. Six themes emerged from the interview findings (1) physical safety and emotional security of babies; (2) parents’ discomfort with babies crying; (3) the role of convenience and practical considerations; (4) their concerns and questions about their babies’ need for separate sleep at some point in development; (5) the influence of whanau; and (6) awareness and reflection on the role of culture in making these decisions. |
| Bauer and Wright, 1996 | United States | Navajo (unknown location) | 35 (Phase I ethnographic interviews); 250 (Phase II, survey interviews); 52 (Phase III, testing revised decision model) | Interviews | The combination of qualitative methods such as decision modeling with statistical analyses provides information about the cognitive and structural factors which influence infant feeding behaviors. Themes highlighted in phase 1: work/job, degree of traditionality, physical inability to breastfeed, believing breast is best, desire to breastfeed, influence of family and health care providers |
| Wright et al., 1993 | United States | Navajo; Kayenta, AZ and Shiprock and Gallup, NM | 35 Navajo men and women, and from survey interviews with 250 postpartum Navajo women. The interviewees included women of childbearing age as well as older, more traditional Navajos. | Interviews and Surveys | Working with Navajo consultants, the authors developed a "cultural text," a  portrayal that synthesized respondents' beliefs. The beliefs highlighted by the text were: that breastfeeding is proper behavior, that it is a means of passing on the mother's attributes, and that it is a means of showing children they are loved. However, only a minority of the ethnographic interviews elicited these beliefs, and only about half the Navajo women surveyed agreed with them as statements. These findings suggest that meanings attached to the body are dynamic, changing over time as aspects of the culturaI context are altered; diverse, reflecting individual experiences and exposure to varied beliefs; and contingent, being influenced by data collection and interpretation technique. |
| MacQuarrie, 1984 | Canada | Ojibway/Anishinaabe (Seven Northwestern Ontario Communities) | 40 women | ethnographic interviews (informal) | Cultural-ideological systems themes: 1) cultural/social value of breastfeeding  2) breastfeeding establishes a close bond between baby and mother  3) Breastfeeding is healthful for the baby  4) breastfeeding is part of a woman's responsibility to her child  5) Value of personal freedom- major reason for not breastfeeding  6) role of grandmother- to allow for more freedom of mother  7) sexuality of female breasts  8) breastfeeding is an "Indian" thing- disconnection from culture and identity  9) milks suitable for native babies- use of carnation milk |
| Horodynski et al., 2012 | United States | 6 Native American Indian communities in a US midwestern state | 42 mothers and 14 health paraprofessionals | Focus groups | The four main themes from the health workers group were: (1) maternal feeding practices for their infants; (2) special considerations when working with the NAI population; (3) sources of advice for NAI mothers; and (4) tailoring a feeding intervention for NAI mothers of infants. Main themes for mothers were: (1) maternal feeding practices for their infants; (2) expectations about an in-home intervention and how to make it work; (3) useful information regarding infant feeding; and (4) importance of family |
| McCalman et al., 2015 | Australia | Muri (Indigenous Australians that traditionally occupied most of modern-day Queensland), Cape York | Seven women who had received Baby Baskets and three family members were interviewed, and 18 health care workers participated in focus groups. The women were either pregnant or recently pregnant, were from six of the eleven Cape York communities and ranged in age from 21 years to 34 years. Family members were aunts who accompanied the women to Cairns for birthing. All but two of the 18 healthcare workers were employed by Apunipima; the others were employed by Mookai Rosie Bi-Bayan Aboriginal hostel which provides accommodation for women awaiting the births of their babies in Cairns. Eight of the healthcare workers were Indigenous (Muri). | Interviews and focus groups | Program implementation entailed empowering families through a process of engaging and relating Murri (Queensland Indigenous) way. Key influencing conditions of the social environment were the remoteness of communities, keeping up with demand, families knowledge, skills and roles and organizational service approaches and capacities. Engaging and relating Murri way occurred through four strategies: connecting through practical support, creating a culturally safe practice, becoming informed and informing others, and linking at the clinic. These strategies resulted in women and families taking responsibility for health through making healthy choices, becoming empowered health consumers, and advocating for community changes. |
| Wright et al., 1997 | United States | Navajo (Shiprock, NM) | 35 for the ethnographic interviews in phase 1 of this project | Interviews and surveys | Cultural beliefs and knowledge impact breastfeeding practices and need to be considered in program development. |
| Abel et al., 2001 | New Zealand | Māori, Tongan, Samoan, Cook Islands, Niuean and Pakeha (European); Auckland, Aotearoa | 150 (27 focus groups plus two one-on-one interviews) | Focus groups | Similarities across all ethnic groups in the perceived importance of breastfeeding and the difficulties experienced in establishing and maintaining this practice. |
| Foley et al., 2013 | Australia | Urban Aboriginal and Torres Straight Islander; Inala, Queensland, Australia | 20 | Interviews | There were five main themes: infant feeding as a socially embedded practice; establishing breastfeeding; mums' feelings about breastfeeding; switching to formula; and addressing feeding challenges |
| Glover et al., 2009 | New Zealand | Māori; Auckland, Palmerston North, Whakatane, Whanganui, and from smaller towns and rural areas in and around Palmerston North and the Bay of Plenty. | 59 Māori women who had given birth in the previous 3 years and 27 whanau (extended family members) | Interviews, Questionnaires with closed and open-ended questions | Although mothers and whanau members feel positive toward breastfeeding and generally expect to breastfeed exclusively, these expectations are unmet in many cases because of lack of support when establishing breastfeeding; lack of support when life circumstances change; lack of timely, culturally relevant, and comprehensible information; confusion about smoking while breast-feeding; uncertainty about the safety of bed-sharing, and perceived lack of acceptability of breastfeeding in public. The relatively high rates of tobacco use by Māori create a tension for breastfeeding mothers, cited by some as a reason for ending breastfeeding prematurely. |
| Glover et al., 2007 | New Zealand | Maori; Auckland and the Bay of Plenty | 41 | Interviews | Many of the women believed that breastfeeding is better for the baby and results in a healthier baby. Practical reasons cited for breastfeeding included being easier, convenient, and free. The following themes were presented: breakdown in the breastfeeding norm within the whanau; early interruptions to or difficulties establishing breastfeeding; negative or insufficient maternity support for breastfeeding; lack of knowledge about how breastfeeding changes over time; and returning to work. |
| Cidro et al., 2015 | Canada | Norway House Cree Nation | 20 interviews and 4 focus groups (# participants unknown) | Interviews and focus groups | Dramatic changes in infant feeding and oral health practices were identified by the research participants as being connected to larger shifts away from cultural-based child-rearing. Breastfeeding and healthy teeth were seen as linked by respondents. The participants were aware that even when it was milk or formula in the bottle, improper feeding techniques and a lack of oral care resulted in tooth decay. The primary themes identified included breastfeeding attitudes, social support for mothers and birthing, supporting healthy infant feeding through community programs, and unhealthy bottle-feeding practices. |
| Cidro et al., 2014 | Canada | Norway House Cree Nation | 20 interview participants and 31 focus group participants | One-on-one interviews and focus groups (which ranged in size from 5 to 10 participants, for a total of 31 focus group participants). | Respondents discussed the importance of feeding infants country food (such as fish, moose and rabbit) at a young age for the overall health of the infant. Related to this was the use of traditional medicine to address oral health issues. Swaddling and other thermal regulation techniques were identified as directly linked to oral health. |
| Kruske et al., 2012 | Australia | Two remote communities in northern Australia | 15 | Interviews and observation | Aboriginal children were highly prized and valued members of a large family network. The children were active agents in determining their own needs, highly prized, and included in all aspects of community life. Themes: Location of the child within the kinship system, cultural practices, and behaviors and beliefs regarding key health topics. |
| Dodgson and Struthers, 2003 | United States | Ojibwe/Chippewa or Anishinabe; Great Lakes region, Northern Minnesota | 44 | Focused ethnographic approach; interviews | Historical factors have negatively impacted breastfeeding; however the majority of women were still aware of traditional practices. The findings are presented in the three categories that emerged from the larger study related to this topic: historical influences, Ojibwe culture, and traditional breastfeeding practices. |
| Dodgson et al., 2002 | United States | Ojibwe; Minneapolis-St-Paul area; 3 rural northern Minnesota reservation communities and 1 urban | 45 | Interviews | There are four patterns that influence breastfeeding including mixed messages, traditions, nurturing and support, and life circumstances. These four patterns identified across group summaries were contextual influences within the social structures of family and community, Ojibwe culture, and mainstream culture. |
| Eni et al., 2014 | Canada | seven First Nation communities (on-reserve) | 65 | “Honest discussions", survey, focus groups | The main findings are that breastfeeding is conducive to bed-sharing, whereas a history of residential school attendance, physical and psychological trauma, evacuations for childbirth, and teen pregnancy are obstacles to breastfeeding. Also, fathers play a pivotal role in the decision to breastfeed. Three overarching themes are discussed: social factors, including perceptions of self; breastfeeding environments; and intimacy, including the contribution of fathers. |
| Moffitt and Dickinson, 2016 | Canada | Tlicho/Dene; Northwest Territories | Chart Audit 2 (n = 198), Chart audit 3 (n = 67), Interviews (n = 8) | Retrospective chart audits, semi-structured interviews | The rate of exclusive breastfeeding initiation in the Tlicho region is less than 30%. Physiological and demographic factors related to breastfeeding were identified. Thematic analysis revealed two overarching themes, namely, the pull to formula (lifestyle preferences, drug and alcohol use, supplementation practices and limited role models) and the pull to breastfeeding (traditional feeding method, spiritual practice and increased bonding with infant). |
| Myers et al., 2014 | Australia | Australian Aboriginal; Victoria | 35 parents and 45 practitioners | Focus groups (parents) and interviews (practitioners) | Three overarching themes are categorized broadly into nutrition concerns, breastfeeding issues and sources of nutrition and child health information and advice. |
| Neander and Morse, 1989 | Canada | Cree; Desmarais, Wabasca and Sandy Lake, AB (and surrounding areas) | 24 | Unstructured, interactive interviews and participant observation (ethnographic research). Informants were interviewed both individually and in multi-generational groups | The major change in childbearing was the removal of childbirth from the household to the hospital. This has resulted in the loss of social support and doulas for mothers, who feel insecure and afraid currently. Infant feeding methods have changed from breastfeeding to the widespread use of canned milk and, if present-day infants are breastfed, it is only for a short time. Both traditional and present-day mothers introduced solid foods early, with traditional mothers chewing the infant’s food rather than using commercial baby food or a blender. Following the traditional practices, mothers felt it was important to keep the breasts warm when lactating. The importance of understanding the cultural context of infant feeding practices is discussed. |
| Helps and Barclay, 2015 | Australia | Australian Aboriginal; 4 rural areas in northern new south wales | 8 | Interviews | The work had three main themes: I'm doing the best thing for...; this is what I know...; and a safe place to feed... The complexities of childhood experiences, historical factors, cultural influences, societal norms and impacts of daily life on infant feeding decisions are discussed. |
| Holmes et al., 1997 | Australia | Australian Aboriginal; Melbourne Metropolitan area from the Victorian Aboriginal Health Service | 7 focus groups and 1 pilot (2-7 in each gorup) | Focus groups | The main themes were: community and psychosocial influences; partner's opinions; health care professionals and hospital practices; beliefs about infant feeding; problems in establishment of breastfeeding |
| Houghtaling et al., 2018 | United States | Assiniboine Bands (Wado-pana and Hudashana) and Sioux Bands (Sisseton, Wahpeton, Yanktonais, and the Teton Hunkpapa); Fort Peck Reservation (northeastern Montana) | 27 grandmothers; 7 health care professionals | Interviews | Main themes: (1) importance of breastfeeding; (2) attachment, bonding, and passing on knowledge; and (3) overburdened health care system. |
| Luby, 2015 | Canada | Anishinabek women; Dalles 28C Reserve, Ontario | ? | Interviews | Hydroelectric power generation on the Winnipeg River disrupted the environment’s ability to provide resources necessary to maintain women's reproductive health (especially breast milk). Food shortages caused by hydroelectric development in the postwar era compromised Anishinabek women's ability to raise their children in accordance with cultural expectations. What emerges from this analysis is a new lens through which to theorize the voluntary enrolment of Anishinabek children in residential schools in northwestern Ontario. |
| Tipene-Leach et al., 2000 | New Zealand | Maori; Auckland, Aotearoa | 26 (17 women and 9 men) | Focus groups and interviews | All parents attempted breastfeeding as it is traditional; however, a variety of factors influenced duration. Main reasons for stopping were cracked and sore nipples and not having enough milk. Main outcomes were not specific to infant feeding; however, themes included: sources of support, customary practices, infant feeding, infant sleeping arrangements and smoking. |
| Weinstein 1999 | United States | Native American; 12 headstart programs | 62 | Interviews | Regarding infant feeding: mothers and caretakers were, for the most part, aware of the risks associated with sleeping with a bottle, it appears that mothers and caretakers with greater parenting experience are more likely to wean at an earlier age; are less likely to put the child to bed with a bottle; and, if the child is put to bed with a bottle, are more likely to engage in activities that minimize risk to the teeth, namely, put water in the bottle, hold the bottle, or remove the bottle, when the child falls asleep. |
| Tapera et al., 2017 | New Zealand | Maori and Samoan; Auckland suburb, Aotearoa | 5 Maori; 2 Samoan | Interviews | Grandparents complementary feeding practices in caring for infant grandchildren were influenced by upstream structural elements such as government policies related to welfare and pensions, employment, income and cultural knowledge. Themes: (i) grandparents understanding of optimal feeding practices; (ii) economic and material factors; (iii) previous experiences and customary norms; and (iv) social support and societal pressure. |
| Cormier, 2014 | Canada | Mi'kmaw, Nova Scotia | Twenty-two women participated in the study conversational interviews and a talking circle | Conversational interviews (open ended questions), a talking circle (focus group), and reflective journaling (done by the author only) | The choice for an infant feeding method was often made out of necessity relating to financial concerns or housing conditions rather than that of a personified belief in either breastfeeding or formula feeding. Findings from the study included four themes: 1) Going it alone—Web of relationships; 2) Finding a space...living in poverty. Is anyone listening?; 3) Is breastfeeding right for me? It’s my choice—respect my choice; and 4) Understanding our time |
| Moffit, 2018 | Canada | Gwich’in • Inuvialuit • Kátł’odeeche • Shúhtaot’ine • Kààlogot’ine • Dehogao’tine • Métis • Dene • Non-Indigenous; four regions of the NWT: Beaufort Delta, Sahtu, DehCho and South Slave regions. | 73 mothers and grandmothers | Sharing circles and semi-structured interview (chart audits for quantitative work) | The 3 overarching themes: Knowledge of infant feeding, Infant feeding today, and Initiation and duration rates of breastfeeding in the NWT. Themes for the Gwich'in sharing circle: feeding practices, being resourceful, surviving hardship, rekindling the past, and sharing wisdom. Themes for the Shuhtaot'ine: being resourceful, surviving hardship, rekindling the past, sharing wisdom, and women and community. Themes for the katlodeeche sharing circle were the same for Gwich'in, although different subthemes. Again, same themes for Inuvialiut sharing circle. Same themes for Fort Smith. Main themes for the interviews were feeding babies, social supports, judgmental discourse, messages to new moms. |
| Helps, 2014 | Australia | Aboriginal; Northern New South Wales | 15- Eight first time Aboriginal mothers, five Aboriginal Health Workers and two community breastfeeding champions. | Semi-structured interviews | Compassionate and holistic maternity care that considers the social and cultural lives of Aboriginal women will be the most effective in supporting them as they make their infant feeding choices. Aboriginal people value and trust knowledge passed to them from extended family members and within their community. Programs to increase the knowledge base of Aboriginal women in the whole community may have greater impact on health outcomes than a reliance on expert health professionals. Three key themes were identified from analysis of the interviews: “I’m doing the best thing for….” which encompasses the expressed and perceived motivations underpinning infant feeding decisions; “this is what I know…” which explores the sources, quality and gaps in knowledge regarding infant feeding; and “ a safe place to feed” identifying the barriers that shame and negative societal messages pose for women as they make infant feeding decisions. An exploration of the impact of historical factors on the Northern NSW Aboriginal community provides a deeper understanding of the cultural context. |
| Moffitt et al., 2018 | Canada | Dene, Métis, Inuvialuit; Northwest Territories | Not described | Elder sharing circles | Preliminary findings from this work challenge the contemporary approach and perceptions of infant feeding practices to combine past and current understandings and values to better support mothers in breastfeeding. Research themes include: resiliency and resourcefulness of mothers, surviving hardships and rekindling past practices. |
| Crosschild, 2019 | Canada | Blackfoot; Southern Alberta | 7 | Group gatherings (information sessions) followed by individual interviews | Main themes: Nurses practicing as Colonial Agents; Resurgent Navigation of Colonial Spaces; and Motherhood as Ceremony. |
| Ross Leitenberger, 1998 | Canada | Northern BC (Dease Lake, Srnithers, Prince Rupert, Houston, Stoney Creek and Terrace) | 7 individual interviews (9 participants total); 8 participants in one focus group; | In-depth interviews and one focus group | Main themes and outcomes: Personal Experiences; Knowledge of Midwifery and Traditional Birthing Practices; Contemporary Birthing. Post analysis, several key patterns or topics emerged within each original heading, producing a more detailed thematic outline. These themes oriented themselves around a holistic way of being. Breastfeeding was a subtheme of Knowledge of Midwifery |
| Gauld, 2009 | Canada | Urban Indigenous women; Thunder Bay, Ontario | 7 Aboriginal women; 7 health workers | In-depth, semi-structured interviews | All mothers in the study were aware of the health benefits of breastfeeding, however most mothers in the study used a combination of breast milk and formula to feed their infants; factors connected to colonial legacy influenced women's feeding decisions; a moral breastfeeding imperative has emerged in Western countries, which can lead to feelings of guilt and alienation. Key categories of findings: infant feeding in everyday life; structural influences; professional influences; the breastfeeding imperative. |
| Goudman, 2014 | Canada | Urban Indigenous women; Edmonton, Alberta | 2 | Narrative inquiry/discussions | Cultural and historical influences profoundly impact a woman’s breastfeeding practices and decisions, and it is necessary to recognize and address these influences to promote breastfeeding in a culturally appropriate manner. Three common threads identified in the stories of the 2 participants: (1) development of an identity and reclamation of culture as central to birthing and breastfeeding experiences; (2) importance of culturally appropriate care based on Aboriginal ways of knowing; (3) importance of recognizing breastfeeding as a natural, spiritual experience, rather than as a sexual, medical experience. |
| Phillips-Beck, 2010 | Canada | Ojibway; Berens River, Manitoba | 31 (14 women and 1 couple in interviews and 15 in 2 sharing circles) | Interviews and sharing circles/focus groups | The researcher divided results into two themes (suffering and hope) and included various subthemes therein. Suffering: emotional impact, the effect on the body, the impact on the family, the effect in the finances, the most significant source of support, the health system in the community, the health system outside the community. Hope: returning birth back to the community, advance preparation in the community, the support in the city, educational support, doula/midwifery support, breastfeeding support, a prenatal only family residence |
| Archibald, 2004 | Canada | Inuit; Nunavut, Labrador, Nunavik, Western Arctic, Ottawa | 20 (structured interviews with Inuit women); 33 in 3 focus groups | Structured interviews and focus groups | Not entirely clear: "The issue of adolescent pregnancy clearly resonated with the people who participated in this study. In addition to concerns for the health and well-being of Inuit children and youth, is a commitment to Inuit culture, values and society. Inuit specific approaches were proposed along with strategies that are consistent with those being promoted in other regions of the country" |
| Wagner, 2005 | Canada | Urban Indigenous; Saskatoon, Saskatchewan | 15 eligible and 8 completed | Interviews, observation with field notes | Results indicated influencing factors are numerous and varied in nature. Contextual (sociocultural and environmental), attitudinal, cognitive (knowledge, information and beliefs), experiential (previous infant feeding experiences), and psychological influences were revealed. |
| Foley and Schubert, 2016 | Australia | Aboriginal and Torres Stright; Brisbane, Queensland | 20 | interviews | Three themes arising from the data are presented here: the impact of care on breastfeeding trajectories, relational support, and mother-centred care. Professional care was found to have a strong influence on breastfeeding initiation and duration. Caring relationships with health professionals which prioritise continuity of care and attention to mothers’ and infants’ specific needs build mothers’ trust in their health carers and their own capacities to breastfeed and in turn facilitate positive breastfeeding outcomes. Mother-centred care in hospital, during clinic follow-up, home visits or phone calls facilitates timely identification of mothers’ feeding doubts or problems and initiation of solutions to breastfeeding problems. Empathic mother-centred infant feeding care is especially important for mothers with limited support at home. |
| Reinfelds, 2015 | New Zealand | Maori and Wahine/Whanau Maori; Taranaki | 11 | Interviews | Thematic analysis revealed many intervention points to support breastfeeding: 1) access to high quality breastfeeding info 2) a compassionaye and culturally safe maternity healthcare workforce 3) active whanau involvement 4) greater acceptance of breastfeeding by wider community 5) access to breastfeeding role models |
| Urban Indian Health Institute, Seattle Indian Health Board, 2011 | US | American Indian and Alaska Native; Detroit, sacramento, salt lake city, and Seattle | 39 (4 focus groups with moms, 1 group with dads, 4 interviews with dads) (27 moms and 12 dads) | Focus groups and discussions | Themes are described in sections related to health and safety for babies: Section 1: “Healthy and Safe Baby” concept Section 2: Health and safety activities and   behaviors  Section 3: Barriers Section 4: Facilitators Section 5: Worries Section 6: Sources of information Section 7: Sources of support Section 8: The role for men/dads Section 9: Communication channels and   messages Section 10: Suggestions  Section 11: Urban life and Urban Indian health   organizations |
| Glover et al., 2006 | New Zealand | Maori; Auckland and the Bay of Plenty | 41 (30 women and 11 family members) | Interviews | Outcomes were grouped as: whanau support for breastfeeding (partners, mothers, sister and aunties, other whanau), how whanau influence infant feeding decisions, whanau perspectives on their role, traditional feeding practices |
| The Victorian Aboriginal Community Controlled Health Organisation (VACCHO), 2012 | Australia | Aboriginal and Torres Straight Islander; Victoria (Mildura, Banyule, Darebin) | 35 (22 male and 13 female) | Focus groups | Key Findings (Parent consultations) 1. Parents in all groups identified sweet drinks, fussy eating and ‘junk’ food as the most common nutrition concerns. Children’s overweight and iron deficiency were mentioned by some parents. 2. Breastfeeding emerged as a dominant issue in both men’s and women’s groups and in both locations. Issues included low rates of breastfeeding, barriers to breastfeeding, attitudes of fathers and an overall lack of ‘culture’ of breastfeeding 3. Barriers to good nutrition identified were lack of appropriate information, lack of nutrition skills, parenting issues, and high food costs. 4. Barriers to physical activity identified were high costs, lack of outdoor play opportunities and reliance on screen-based activities. 5. The most frequently reported systemic issue was lack of continuity of care in early childhood for example; access to MCH services. 6. Aboriginal parents were more likely to experience difficulties accessing nutrition and physical activity information and support than non-Aboriginal parents. 7. Food insecurity and other, broader social determinants of health were frequently reported. |
| Martens, 1999 | Canada | First Nations/Anishinaabe/Ojibway; Sagkeeng First Nation, Manitoba | 22 interviews for PC program evaluation (13 PC clients; 9 non-clients) | Interviews, surveys and chart audits | For all women- 4 themes for benefits: bonding, healthiness of baby, ease, and convenience and less costly; 3 themes for problems: soreness, insufficient milk, and latching. The qualitative interviews  indicated that PC clients had greater access to information, more satisfaction with their breastfeeding experience because of this access, and less problems due to timely answers to their concerns. In contrast, non-clients referred to the need for a good source of information. |
| Oneha and Dodgson, 2009 | US/Hawaii | Native Hawaiian; Wai'anae, on O'ahu | 20 | Interviews | The patterns were identifying resources, difficulty breastfeeding, and unmet expectations. Resources which functioned as supports or assets to the breastfeeding experience included knowledge, prior experience, motivation, support, equipment, and empathy. Breastfeeding was “hard,” because of the physical problems with the woman’s breast, discomfort, and time demands. Unmet expectations included a discrepancy in values, an assumption of participant knowledge, lifestyle adjustments, and the value of organizational support. |
